# The Impact of DNA Methylation in *Trichoderma reesei* on Cellulase Production and Strain Degeneration

**DOI:** 10.3390/microorganisms13030584

**Published:** 2025-03-04

**Authors:** Caroline Danner, Thiago M. Mello de Sousa, Robert L. Mach, Astrid R. Mach-Aigner

**Affiliations:** 1 Christian Doppler Laboratory for Optimized Expression of Carbohydrate-Active Enzymes, Institute of Chemical, Environmental and Bioscience Engineering, TU Wien, Gumpendorfer Str. 1a, 1060 Vienna, Austria; caroline.danner@tuwien.ac.at (C.D.);; 2 Institute of Chemical, Environmental and Bioscience Engineering, TU Wien, Gumpendorfer Str. 1a, 1060 Vienna, Austria; robert.mach@tuwien.ac.at

**Keywords:** cellulase, DNA methylation, DNA methyltransferase, *Trichoderma reesei*, strain degeneration

## Abstract

The spontaneous loss of cellulase productivity of industrial *T. reesei* strains during production results in significant economic losses. This phenomenon was suggested to be epigenetically regulated, but the previous findings did not explain which epigenetic mechanisms occur and how they promote strain degeneration. Until now, the epigenetic landscape of *T. reesei* has been poorly understood. This study investigated whether DNA methylation and cellulase production are connected, and, if so, what that connection is and how it relates to strain degeneration. In order to determine what the impact of DNA methylation is on strain degeneration, we induced hypomethylation with hydralazine HCL, which showed a reduced non-productive phenotype and partially restored cellulase productivity. As a second test, we conducted a global DNA cytosine methylation assay, which showed *T. reesei* DNA methylation levels of between 0.2 and 1.3% 5-mC. Importantly, non-productive strains exhibited stronger methylation than productive counterparts, and global methylation patterns varied depending on the carbon source. As a final test, we carried out deletion experiments targeting the putative DNA methyltransferases *Dim2* and *Rid1*, which initially reduced the occurrence of a non-producing subpopulation, but subsequent sub-cultivation eliminated cellulase productivity. This study shows that DNA methylation impacts *T. reesei* cellulase productivity, an understanding that can help us develop targeted strategies to reduce strain degeneration and improve cellulase production in industrial applications.

## 1. Introduction

In nature, the filamentous fungus *Trichoderma reesei* secretes enzymes to degrade plant biomass. These carbohydrate-active enzymes, such as (hemi)cellulases or xylanases, are used in an industrial context for numerous applications. Cellulases are applied, for example, for recycling processes in the paper industry, in the textile industry for fiber polishing, and for bioethanol production from lignocellulosic biomass [1]. According to the current understanding, cellulase expression is mainly regulated transcriptionally in response to the available carbon source. The transcription is de-repressed under glucose-limiting conditions. The presence of an inducer, for example, cellulose and ß-linked disaccharides such as cellobiose, sophorose, or lactose, fully activates cellulase production [2,3]. The cellulase encoding genes are normally subjected to carbon-catabolite repression (CCR) mediated by the zinc finger protein Cre1 (carbon catabolite repressor 1) [4,5]. Substantial work in strain improvement through mutagenesis and screening led to hyperproductive *T. reesei* strains with an outstanding secretion capacity of up to 100 g/L [6]. The industrial strains Iogen-M4 [7] and Iogen-M10 [8] used in this study were derived from the hypercellulytic strain Rut-C30 [9], a descendant of the wild-type *T. reesei* strain QM6a. Rut-C30 and derived strains lack the full length of Cre1 and, therefore, are partially de-repressed under glucose conditions [10].

The industrial exploitation of hyperproductive strains has negative consequences, which is the spontaneous loss of productivity. It was reported that during the fed-batch process of the industrial production of cellulase, hyperproductive *T. reesei* strains Iogen-M4 and Iogen-M10 spontaneously lose productivity [8]. This phenomenon is called spontaneous degeneration and refers to a loss of or decline in the production capacity of a microorganism. It is a widespread occurrence in the biotechnical industry, impacting numerous microorganisms used as hosts for various products and causing substantial economic losses. Other biotechnologically significant filamentous fungi, including *Aspergillus* and *Penicillium*, have also exhibited spontaneous declines in productivity [11]. For instance, *Aspergillus niger* has been reported to experience a reduction in citric acid and total organic acid productivity, while *Penicillium chrysogenum* has shown a decrease in penicillin productivity [12,13,14,15]. The mechanisms driving fungal degeneration can be genetic or epigenetic, with additional factors such as stress, aging, or culture conditions also contributing [11]. Understanding the underlying causes of degeneration is crucial for ensuring production stability in industrial settings.

To study strain degeneration in *T. reesei*, a lab-scale artificial degeneration protocol, hereafter referred to as induced strain degeneration (ISD) protocol, was designed [8]. The ISD yields a parameter known as the degeneration rate, representing the proportion of the non-cellulase producing (cel-) subpopulation relative to the entire population. The degeneration was found to be connected to the productivity of the strain. The highly productive strains such as Iogen-M10 showed a higher degeneration rate (around 90–100%) than moderate producers; for example, Iogen-M4 (20–60%) or Rut-C30 (10–20%) and the wild-type QM6a hardly degenerate (0–10%) [8].

Up to now, no full explanation has been found on what causes this drastic loss of cellulase productivity in *T. reesei*. It has been suggested that the degeneration phenomenon is more likely regulated by epigenetic rather than genetic mechanisms due to three reasons. First, there is a gradual development from a semi (cel-) to a full (cel-) phenotype, which is not reversible and heritable. Secondly, trichostatin A, an inhibitor of heterochromatin formation, reduced the accumulation of a (cel-) population at an earlier time point. Thirdly, higher chromatin compaction was found in the promotor regions of the main cellulase-related genes in the (cel-) strains, compared to the productive strains, under inducing and non-inducing conditions [8]. Together, this has shown that epigenetic mechanisms are likely to be involved in the degeneration phenomenon of *T. reesei.* What is unknown is what epigenetic mechanisms are involved and how they influence the spontaneous loss of cellulase productivity.

In general, knowledge of the epigenome, including DNA and chromatin modifications in *T. reesei,* is scarce [16]. Yet, past research has shown that the production of cellulase is linked to the regulation of the chromatin structure. In particular, the regulation of cellulase gene expression was found to be connected to chromatin remodeling and nucleosome positioning [17,18,19,20]. The two important transcription factors, Cre1 and Xyr1 in *T. reesei*, are reported to contribute to chromatin remodeling by influencing the position of nucleosomes in promotor or coding regions of cellulase-related genes [17,18,20,21,22,23]. Histone acetylation, in addition, appears to influence cellulase expression [21]. This is important for understanding that epigenetic mechanisms are part of the complex regulation of cellulase expression. What we do not know yet is if and what other epigenetic mechanisms are involved in the regulation of cellulase production.

We considered it essential to investigate if the degeneration phenomenon is connected to DNA methylation because the formation and inheritance of heterochromatin and DNA methylation are frequently associated. So far, there is no report on the existence of DNA methylation in *T. reesei,* and knowledge on its regulation and its influence on cellulase production is currently lacking. Many fungi have lost or degraded their DNA methylation machinery, even though DNA methylation is essential for various nuclear processes in other organisms [24,25]. The initial research on DNA methylation in fungi was mainly studied in *Neurospora crassa*. The regulation of methylation involves two primary aspects: the maintenance of existing methylation patterns and the establishment of de novo methylation marks, typically orchestrated by different proteins [26]. DNA methyltransferases (DMTs) play a key role in methylating cytosine residues [25,27]. In *N. crassa*, a single DNA methyltransferase, Dim2 (Defective in Methylation 2), is essential for all known DNA methylation. Maintenance methylases inherit DNA methylation marks [28]. Additionally, some methylation marks can lead to DNA mutations, particularly within the genome defense mechanism RIP (Repeat-Induced Point Mutation). In *T. reesei*, the orthologues of Rid1 (RIP defective 1) and Dim2 have been implicated in the RIP process [29,30].

For the first time, this study offers a thorough investigation into DNA methylation across diverse cellulase-producing *T. reesei* strains. The aim was to explore whether DNA methylation impacts cellulase production and, in particular, to investigate its potential correlation with strain degeneration. We studied whether DNA methylation leads to strain degeneration and found that it does in a two-step process. Firstly, through artificially inducing hypomethylation, we observed a reduction in the (cel-) population and a subsequent increase in cellulase activity in (cel-) strains. Secondly, we explored the impact of deleting two putative DMT-encoding genes, *dim2* and *rid1*, on the degeneration phenomenon and cellulase production. These findings contribute to a deeper comprehension of the degeneration phenomenon because they show that DNA methylation is increasing the formation and retention of the non-productive phenotype. In addition, our findings, showing that DNA methylation in *T. reesei* potentially regulates cellulase production, help us better understand the influence of epigenetic mechanisms in cellulase regulation.

## 2. Materials and Methods

### 2.1. Fungal Strains

The following *T. reesei* strains were used for this study: the wild-type strain QM6a (ATCC 13631); the strain Rut-C30, which is described as hypercellulolytic and was derived from three rounds of mutagenesis and screening from QM6a, (ATCC 56765) [31]; the industrially used strains Iogen-M4 [7] and Iogen-M10 [8] (proprietary of Novozymes, Bagsvaerd, Denmark); their non-productive counterpart strains Iogen-M4 (cel-) and Iogen-M10 (cel-) [8] (proprietary of Novozymes, Bagsvaerd, Denmark). These strains were chosen due to their differences in their capacity to produce cellulases and the potential different reasons for this circumstance (genetic versus epigenetic mechanisms). A short description of the characteristics of those strains can be found in Table 1. Further, the *dim1* and *rid2* deletion strains of Iogen-M10 (this study; proprietary of Novozymes, Bagsvaerd, Denmark) were investigated. The strains were maintained on potato dextrose agar plates or on malt extract (MEX) plates (3% (*w*/*v*) malt extract, 0.1% (*w*/*v*) peptone, and 1.5% (*w*/*v*) agar). For short-term storage, the strains were kept on plates at 4 °C and, for long-term storage, as spore suspensions in 25% glycerol at −80 °C.

### 2.2. Growth Conditions

For the carbon source replacement experiments, mycelium was pre-cultured in 1 L Erlenmeyer flasks at 30 °C and 180 rpm for 24 h in 250 mL of Mandels-Andreotti (MA) medium [33] supplemented with 1% (*w*/*v*) glycerol as sole carbon source. A total of 10^9^ spores per liter (final concentration) were used to inoculate. Equal amounts of washed, pre-grown mycelium were transferred to 20 mL MA medium containing 1% (*w*/*v*) D-glucose, 2 mM sophorose, or 0.5 mM xylose as a sole carbon source or no carbon source and further incubated for 3 h.

For direct cultivations, spores were incubated in 200 mL MA medium with 1% (*w*/*v*) lactose or 1% (*w*/*v*) D-glucose as the sole carbon source for 48 h. For the induction of hypomethylation, hydralazine-HCl in concentrations of 0 µM, 250 µM, or 1000 µM was added to the MA medium. Samples were derived from three biological replicates.

### 2.3. Construction of Deletion Cassettes for dim2 and rid1

Deletion cassettes for *dim2* and *rid1* with homologous flanks were constructed as described in [34]. PCRs for cloning purposes were performed with the Q5 High-Fidelity DNA polymerase (New England Biolabs, Ipswich, MA, USA), according to the manufacturer’s instructions. Cloning was conducted using the pJET1.2 vector system (Thermo Scientific, Waltham, MA, USA) following the manufacturer’s guidelines.

For the construction of *rid1* deletion vector pTSΔrid1_hph (amp), 3′ and 5′ homology flanks were cloned in pJET1.2 and a hygromycin resistance cassette was inserted between the 5′ and 3′ flanking regions. For the 3′ and 5′ flank of *rid1*, two PCR products were created with the primers TS101_rid1(BamHI) and TSI02_rid1 or TS99_rid1(NotI) and TS100_rid (BamHI), respectively, using the genomic DNA of Rut-C30 as a template. The flanks and the hygromycin resistance cassette were inserted into pJET1.2 using restriction enzyme digestion by *Not*I and *BamH*I and subsequent ligation.

For the construction of the *dim2* deletion vector pTSΔdim2_hph (amp), the same strategy was followed as described for *rid1*. The primers TS54 (NotI) and TS55 (internal *BamH*I restriction site available) or TS56 (BamHI) and TS57 were used to construct the 5′ flank and 3′ flank, respectively. The primer sequences can be found in Table S1, and the vectors are figuratively shown in Figure S1 in the Supplementary Material.

### 2.4. Fungal Transformation

Transformation of *T. reesei* protoplasts was performed, as described by [34,35]. Usually, 30 to 50 µg of digested plasmid DNA dissolved in 15 µL sterile double-distilled water was used to transform 10^7^ protoplasts in a total volume of 200 µL. Further, 100 µL to 2 mL of the transformation reaction mixture was added to 10 mL molten, 50 °C warm, MEX agar containing 1.2 M sorbitol. Then, the mixture was poured into sterile petri dishes and incubated at 30 °C for 5 h. After solidification, an overlay of 10 mL molten, 50 °C warm MEX agar containing 1.2 M sorbitol and 200 µg/mL of hygromycin B was poured onto the protoplast-containing layer. Plates were incubated at 30 °C for 2 to 5 days until colonies were visible.

### 2.5. Genotyping of Deletion Strains

The deletion of *dim2* and *rid1* was verified by PCR using two sets of primers for the presence of the wild-type gene and the integration of the hygromycin marker gene *hph* in the correct locus. The primer sequences and the result of the gel electrophoresis can be found in Table S1 and Figure S2 of the Supplementary Material. To verify if there is no transcription of *dim2* or *rid1*, the deletion strains were grown for 120 h in MA medium containing 1% (*w*/*v*) lactose at 30 °C and 160 rpm, and then mycelium was harvested. RNA extraction, cDNA synthesis, and qPCR were performed, as described in Section 2.9. The qPCR primer sequences can be found in Table S1 of the Supplementary Material.

### 2.6. Induced Strain Degeneration (ISD) Protocol

The protocol developed by Martzy and colleagues was used for the lab-scale, artificial degeneration of *T. reesei* strains [8]. *T. reesei* strains were inoculated to 2 × 10^7^ spores in 20 mL of MA medium containing 1% (*w*/*v*) lactose and 0.1% (*w*/*v*) peptone and incubated for 120 h at 30 °C and 160 rpm. For the hydralazine treatment, 1 mM hydralazine-HCl was added to the MA medium. Then, mycelium was harvested and regenerated on MEX plates via incubation at 30 °C for 5 days until sporulation. Obtained spores were then used for the determination of cellulase activity.

### 2.7. Cellulase Activity Assay

The cellulase activity of the samples from the ISD protocol was determined by spreading out 100–200 spores on plates containing MA medium with 0.5% (*w*/*v*) CMC (carboxymethylcellulose sodium salt, high viscosity), 0.1% (*w*/*v*) peptone and 2.0% (*w*/*v*) agar. After incubation at 30 °C for 3 days, the plates were incubated for 4 h at 50 °C. Then, the plates were flooded with 10 mL of Congo red dye (2.5 g/L) and stained for 15 min at room temperature with gentle stirring, followed by a washing step with 10 mL of 1 M NaCl. The cellulase secretion and activity are indicated by the formation of a clearing zone around the colony. The percentage of (cel-) occurrence was determined by counting the cellulase-producing and non-producing colonies.

The cellulase activity in liquid culture was measured using the Cellazyme C Tablets (Megazyme, Wicklow, Ireland) following the manufacturer’s instructions (40 °C for 10 min). One unit of activity is defined as the quantity of enzyme necessary to release one micromole of glucose reducing-sugar-equivalents per minute. Samples from three biological replicates and two technical duplicates were measured. To measure biomass (dry weight), the mycelia were harvested through filtration, washed with an equal volume of 0.8% NaCl solution, dried for 24 h at 80 °C, and weighted. Values are presented in units/mL * g biomass.

### 2.8. Global DNA Methylation Assay

Fungal mycelium was homogenized in 1 mL CTAB buffer (100 mM TRIS-HCl pH = 8, 1.4 M NaCl, 20 mM EDTA, 2% (*w*/*v*) CTAB) using a FastPrep(R)-24 cell disrupter (MP Biomedicals, Santa Ana, CA, USA). Chromosomal DNA was extracted in two rounds of phenol-chloroform extraction [35]. DNA concentration was measured using the NanoDrop 1000 (Thermo Scientific, Waltham, MA, USA).

The global DNA methylation pattern was quantified with the MethylFlash Methylated DNA quantification kit (P-1034, Epigentek, Farmingdale, New York, NY, USA), according to the manufacturer’s instructions, using 100 ng of genomic DNA as starting material. The samples were measured in three biological replicates and technical duplicates.

### 2.9. Transcript Analysis

RNA was extracted from fungal mycelium according to the manual instructions through homogenization in 1 mL of peqGOLD-TriFast DNA/RNA/protein purification system reagent (PEQLAB Biotechnologie, Erlangen, Germany) using a FastPrep(R)-24 cell disrupter (MP Biomedicals, Santa Ana, CA, USA). RNA concentration was measured using the NanoDrop 1000 (Thermo Scientific, Waltham, MA, USA). Synthesis of cDNA from mRNA was performed with the RevertAidTM H Minus First Strand cDNA Synthesis Kit (Thermo Fisher Scientific) following the manufacturer’s instructions. Quantitative PCRs were conducted in triplicates in a Rotor-Gene Q system (Qiagen, Hilden, Germany). The amplification mixture, with a final volume of 15 μL, comprised 7.5 μL of 2× iQ SYBR Green Mix (Bio-Rad, Hercules, CA, USA), 100 nM each of forward and reverse primers, and 2.5 μL of cDNA (diluted 1:20) as template. The primer sequences can be found in Supplementary Material Table S1. Cycling conditions and control reactions were executed following the procedures outlined in [36]. Data normalization utilized *sar1* and *act* as reference genes, and the calculations were carried out as detailed in [36].

### 2.10. Statistical Analysis

The significance of the differences observed between multiple experimental groups in this study was assessed using a one-way analysis of variance (ANOVA) followed by a post hoc pairwise comparison. The ANOVA tested for significant differences in the groups by computing the between-group and within-group sum of squares, plus the mean squares and F-statistic. Then, to specifically identify differences between group means, t-tests with pooled variance were used to calculate the post hoc pairwise comparisons. For each comparison, *p*-values were calculated and corrected for multiple comparisons with the Benjamini–Hochberg method. Significance thresholds were set to *p* < 0.05, with significance levels indicated as *** (*p* < 0.001), ** (*p* ≤ 0.01), * (*p* ≤ 0.05), and (*p* ≤ 0.1). All statistical analyses were performed in R studio version R.4.3.1.

## 3. Results

### 3.1. DNA Hypomethylation Partly Prevents the Degeneration Phenomenon

To investigate the potential role of DNA methylation in the spontaneous loss of cellulase productivity, we first explored the effect of hypomethylation on the strain degeneration phenomenon. The chemical agent hydralazine is reported to cause DNA hypomethylation and to interact with DNA methyltransferases [37,38,39,40,41]. In order to determine whether the occurrence of a (cel-) subpopulation changes upon hypomethylation, we subjected the two industrially used strains, Iogen-M4 and Iogen-M10, to the ISD protocol in the presence and absence of hydralazine-HCl.

**Figure 1:**
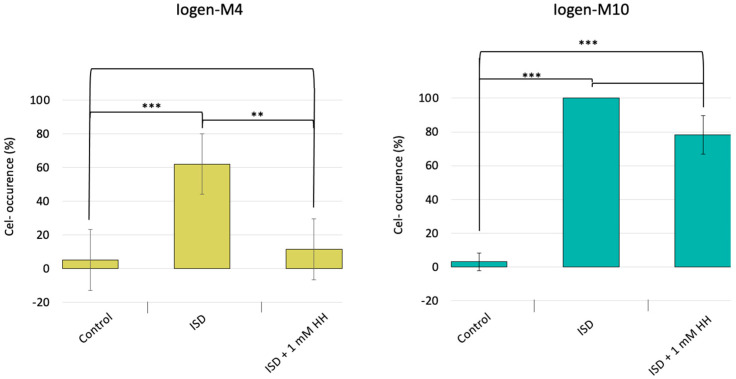
The (cel-) occurrence in dependence of hypomethylation. The hypercellulase-producing strains Iogen-M4 and Iogen-M10 were subjected to the ISD protocol without and with the addition of 1 mM hydralazine HCl (HH). The occurrence of the (cel-) population is given as a percentage before (control) and after the ISD protocol. The displayed mean values were obtained from three biological replicates; error bars indicate standard deviations of the means, and the significance levels are indicated as *** (*p* < 0.001), ** (*p* ≤ 0.01), * (*p* ≤ 0.05), and (*p* ≤ 0.1).

As observed earlier, the degeneration is more pronounced in the highly productive strain Iogen-M10, showing a (cel-) occurrence of 100% after the ISD protocol, compared to approximately 60% for the moderately productive Iogen-M4 (Figure 1). Notably, the occurrence of the (cel-) population is reduced for both strains due to the addition of hydralazine. The impact is more pronounced in Iogen-M4, where hypomethylation leads to an impressive reduction in the degeneration rate by approximately 80%.

**Figure 2:**
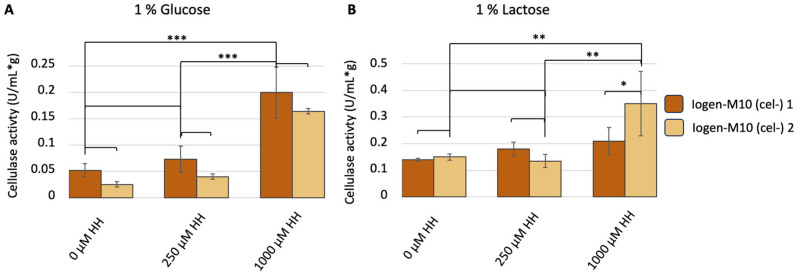
Cellulase activity dependent on hypomethylation. Two Iogen-M10 (cel-) strains (dark and light-brown bars) were cultured in MA medium with (**A**) 1% D-glucose or (**B**) 1% lactose as the sole carbon source and the indicated concentration of hydralazine-HCl (HH) for 48 h. One unit of activity determined in the supernatant is defined as the quantity of enzyme necessary to release one micromole of glucose reducing-sugar-equivalents per minute and normalized to the biomass (g). The displayed mean values were obtained from three biological replicates; error bars indicate standard deviations, and the significance levels are indicated as *** (*p* < 0.001), ** (*p* ≤ 0.01), * (*p* ≤ 0.05), and (*p* ≤ 0.1).

In a second experiment, the eventual reversibility of a degenerated population by hypomethylation was investigated. Two Iogen-M10 (cel-) strains were cultivated under cellulase-inducing (lactose) and non-inducing (glucose) conditions in the absence and presence of hydralazine. The addition of hydralazine led to an increase in cellulase activity in all tested conditions (Figure 2).

The increase in cellulase activity correlates positively with the hydralazine concentration. This was the first approach that resulted in a partial reversion of the (cel-) phenotype, though the levels of the productive parent strain (10 ± 2 U/mL*g on glucose, 54 ± 1 U/mL*g on lactose) could not be reached.

### 3.2. Global Methylation Pattern of Differently Productive T. reesei Strains

**Figure 3:**
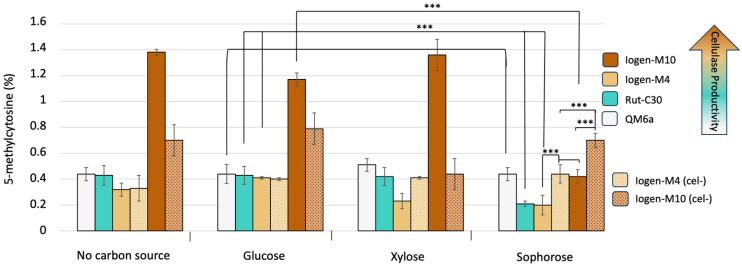
Global methylation of DNA in *T. reesei* strains. The strains (color code provided) were pre-cultured and transferred to MA medium containing D-glucose, D-xylose, or sophorose, and a medium containing no carbon source as a reference. The cellulase productivity of the strains is indicated by the provided color code. The percentage of 5-methyl cytosine (5-mC) was determined by an immunoenzymatic assay using the MethylFlash kit (Epigentek). The displayed mean values were obtained from three biological replicates, error bars indicate standard deviations and the significance levels are indicated as *** (*p* < 0.001), ** (*p* ≤ 0.01), * (*p* ≤ 0.05), and (*p* ≤ 0.1).

To further understand the role of DNA methylation in the strain degeneration phenomenon and cellulase expression of *T. reesei*, we compared the global methylation pattern of differently productive strains (the wild-type QM6a, Rut-C30, industrial strains Iogen-M4 and Iogen-M10 as well as their (cel-) counterparts) in the presence of different carbon sources. The cellulase productivity of these strains is described in the study [8]. Generally, the DNA of *T. reesei* was found to be methylated to an extent of 0.2 to 1.3% 5-mC and varied depending on the strain and the carbon source (Figure 3).

The hypercellulase-producing strains Rut-C30 and Iogen-M4 have a reduced degree of methylation in cellulase-inducing conditions (sophorose) compared to the methylation pattern of the wild-type QM6a. The latter has a similar extent of DNA methylation, regardless of the carbon source. Importantly, the most productive strain, Iogen-M10, had a similar degree of methylation in the absence of a carbon source as in the presence of D-glucose or D-xylose but the lowest degree of DNA methylation on the cellulase-inducing sophorose. Notably, the degree of DNA methylation is higher for both (cel-) strains compared to the productive counterpart on sophorose. This is not the case for the condition without any carbon source or with D-glucose. In the xylanase-inducing condition (D-xylose), Iogen-M4 (cel-) also shows a higher methylation degree compared to the productive strain, yielding a similar pattern as for sophorose. This is not the case for Iogen-M10, which was strongly selected for improved cellulase expression.

### 3.3. The Role of the Putative DNA Methyltransferases Dim2 and Rid1 in Strain Degeneration

To investigate the effect of the two putative DMTs, Dim2 and Rid1, on strain degeneration, we decided to delete their encoding genes from the genome of Iogen-M10 and to subject the resulting strains to the ISD protocol. Genotyping by PCR confirmed the successful deletion of the genes (Figure S2, Supplementary Material). To verify the successful deletions, the transcript levels of *dim2* and *rid1* of the Δ*dim2* and the Δ*rid1* strain were compared to the parent strain. For this purpose, the strains were cultured in liquid culture for 120 h, and the relative transcript ratios were determined by RT-qPCR. As expected, no *dim2* transcript was found in the Δ*dim2* strain, while it could be detected in the parent strain. Otherwise, despite multiple attempts to optimize the PCR, no transcript of *rid1* could be detected in either the Iogen-M10 strain or the Δ*rid1* strain.

**Figure 4:**
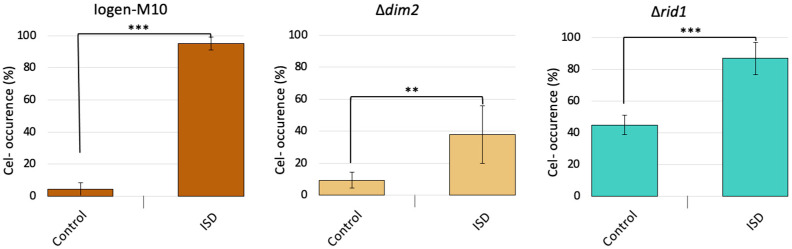
The (cel-) occurrence (%) dependent on the deletion of the DNA methyltransferases Dim2 and Rid1. The hypercellulase-producing strain Iogen-M10 and the *dim2* and *rid1* deletion strains were subjected to the ISD protocol. The occurrence of the (cel-) population is given as a percentage before (Control) and after the ISD protocol. The displayed mean values were obtained from three biological replicates and error bars indicate standard deviations and the significance levels are indicated as *** (*p* < 0.001), ** (*p* ≤ 0.01), * (*p* ≤ 0.05), and (*p* ≤ 0.1).

The results of the ISD protocol showed that deleting *dim2* strongly reduced the occurrence of the (cel-) phenotype from around 90% to 37%, indicating a positive effect of the absence of Dim2 on cellulase production stability. In contrast, the deletion of *rid1* resulted in an 87% degeneration rate that is similar to the parent strain Iogen-M10 (Figure 4).

**Figure 5:**
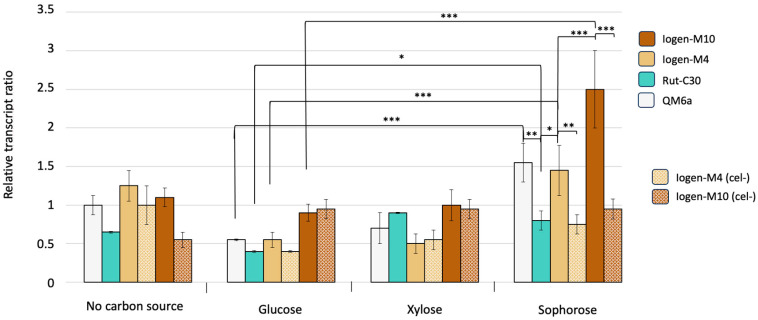
Transcript levels of *dim2* in *T. reesei* strains. The strains (color code provided) were precultured and transferred to MA medium containing D-glucose, D-xylose, or sophorose and to a medium containing no carbon source as a reference. The relative transcript ratios were determined by RT-qPCR. Data were normalized to *act* and *sar* genes and QM6a on no carbon source serves as the reference condition. The displayed mean values were obtained from three biological replicates and error bars indicate standard deviations and the significance levels are indicated as *** (*p* < 0.001), ** (*p* ≤ 0.01), * (*p* ≤ 0.05), and (*p* ≤ 0.1).

In a further exploration, to assess the potential influence of the putative DMT Dim2 on cellulase productivity, we examined its expression across diverse *T. reesei* strains and under different carbon sources. The transcript levels of *dim2* are shown in Figure 5. Generally, *dim2* seems to be upregulated in cellulase-inducing conditions (sophorose) in the strains QM6a, Iogen-M4, and Iogen-M10 compared to the other conditions. The expression of *dim2* on sophorose is reduced in the (cel-) strains compared to their productive counterparts. This difference is higher for the more productive strain Iogen-M10.

**Figure 6:**
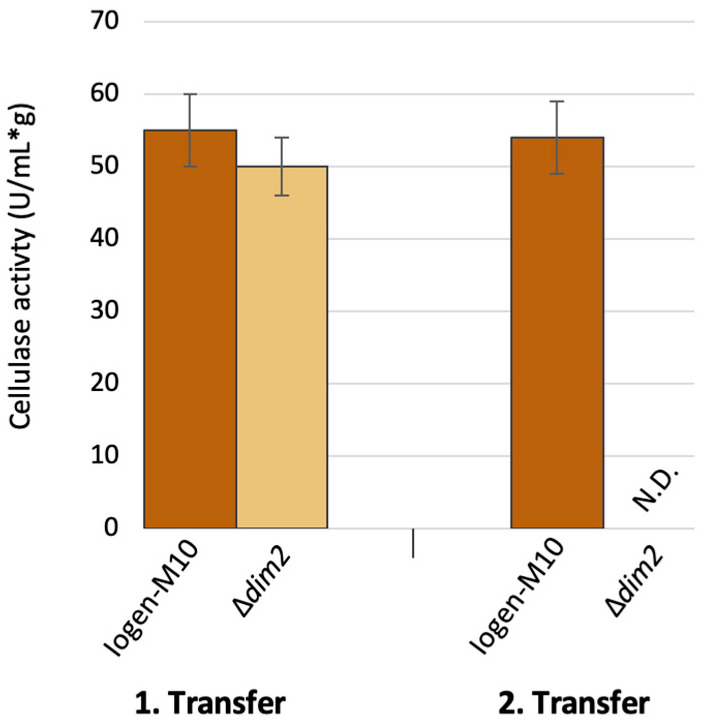
Cellulase activity of the *dim2* deletion strain (light brown) compared to the parent strain Iogen-M10 (dark brown). The strains were cultivated on cellulase inducing conditions (Cultivation 1), regenerated on MEX and then cultivated again on cellulase inducing conditions (Cultivation 2). One unit of activity is defined as the quantity of enzyme necessary to release one micromole of glucose reducing-sugar-equivalents per minute and normalized to biomass (g). The displayed mean values were obtained from three biological replicates and error bars indicate standard deviations. N.D., not detected.

As the deletion of *dim2* promised a certain prevention of the strain degeneration phenomenon, the stability of this effect was tested. Therefore, a repeated cultivation of the strain in cellulase-inducing conditions was performed. After the first cultivation, the cellulase activity of the Δ*dim2* strain was comparable to its parent strain Iogen-M10 (Figure 6). After the regeneration of the strain on full medium (i.e., MEX) and another round of cultivation in cellulase-inducing conditions, no cellulase activity could be detected for the Δ*dim2* strain.

## 4. Discussion

In this study, we explored the relationship between DNA methylation and cellulase expression in *T. reesei*, with a special focus on the role of DNA methylation in the phenomenon of the spontaneous degeneration of cellulase productivity. For the first time, we demonstrate that DNA methylation is occurring in *T. reesei* at all and that it is involved in the regulation of cellulase expression. An analysis of the global DNA methylation pattern in different *T. reesei* strains revealed dynamic changes in response to different carbon sources. The hypercellulase-producing strains Rut-C30, Iogen-M4, and Iogen-M10 displayed reduced DNA methylation under cellulase-inducing conditions compared to non-inducing conditions. The most significant change was found for the most productive strain, Iogen-M10. The DNA methylation status of this strain seems to be intricately linked to the carbon source availability, suggesting a regulatory role of DNA methylation in response to cellulase induction conditions. This aligns with the fact that the regulation of cellulase gene expression is associated with chromatin remodeling and accessibility, which has been reported to be dependent on the available carbon source as well [17,18,20,22]. Interestingly, it was found that both a more open or closed chromatin status can be more sensitive towards methylation [21]. The study of Martzy and colleagues demonstrated that (cel-) strains have more compact chromatin. They suggested that the heterochromatin formation in (cel-) strains may be involved more in the development rather than the initiation of the degeneration [8]. We found in this study that the non-cellulase-producing phenotype displays a greater level of methylation compared to its productive counterpart. A higher degree of methylation is usually connected to lower gene expression. This explains the non-reversibility of the loss of cellulase production to some extent. Connecting this finding with the reported enriched level of heterochromatin in cellulase-related genes suggests that DNA methylation could induce the degeneration phenomenon and could further induce the formation of heterochromatin, ultimately resulting in a lower expression of cellulase genes. However, since neither the hypomethylation nor the deletion of the putative DMT Dim2 prevented the strain degeneration entirely, it needs to be considered that there might be additional regulatory mechanisms involved. Nevertheless, the initiation, development, and inheritance of the non-productive phenotype might be regulated through de novo DNA methylation and its maintenance [8].

It has to be noted that this study focused on the change in global DNA methylation in regard to solely cytosine methylation. However, there are other less studied forms of DNA methylation, such as 5-hydroxymethylcytosine (5hmC), N6-methyladenine (6mA), or N4-methylcytosine (4mC), known to have important roles in the biological processes of model organisms and humans [26]. To draw more concrete conclusions on the regulatory mechanism of DNA methylation in connection to the loss of cellulase productivity, the next step could be to specifically analyze methylation marks at the nucleotide level. This would allow us to identify specific genomic regions in which DNA methylation is enriched and find methylation marks that could be used as a target for the prevention of this phenomenon.

In addition, we found that the putative DMT Dim2 has a regulatory role in cellulase production, while Rid1 is likely inactive in *T. reesei*. The finding that certain methyltransferases, including putative DMTs, are involved in cellulase regulation aligns with previous reports in the literature. The deletion of *lae1*, the *T. reesei* ortholog of the *A. nidulans laeA*, led to a decrease in *xyr1* expression and reduced cellulase production [42]. Although Lae1 was initially postulated as a putative protein methyltransferase, recent studies indicate that its function is not linked to histone or protein methylation. Instead, LaeA/Lae1 undergoes self-methylation, but this modification is hypothesized not to be essential for its regulatory role [43]. Also, the deletion of the putative DNA methylation modulator-2 (Dmm2) enhanced cellulase production by 20–30% in *T. reesei* [44]. In *Penicillium oxalicum*, different LaeA-like methyltransferases had an effect on the expression of cellulase-encoding genes [45]. Therefore, DMTs or other DNA methylation modulators could be possible targets for future strain improvements. In turn, it has to be kept in mind that the *dim2* deletion strain used in this study again lost cellulase activity after sub-cultivation. There are numerous possibilities as to why the deletion of *dim2* leads to this loss of cellulase activity. A disruption of the methylation pattern of the strain could ultimately affect the epigenetic regulation of cellulase expression, including transcription factor binding, chromatin structure, and gene accessibility. In addition, genome integrity could be compromised, leading to cellular stress that deprioritizes cellulase production. In contrast, the deletion of *rid1* had no effect on the stability of cellulase productivity, and *rid1* expression could not be detected. Interestingly, in other ascomycetes, such as *N. crassa*, *Magnaporthe oryazae* and *Verticillium dahliae*, Rid1 was found to have no or only minor DNA methylation activity [26,46,47]. Further, Rid1 was associated with being active only in the sexual cycle of *N. crassa* [46]. The industrial *T. reesei* strains are asexual, which could be a possible explanation for why, in this study, no *rid1* transcript could be detected. Further studies are needed to verify or falsify the presence and activity of Rid1 in *T. reesei.* At first sight, a contradicting observation is that Iogen-M10 (cel-) on sophorose has higher DNA methylation but shows a lower *dim2* transcript than Iogen-M10. This suggests that the expression or activation of Dim2 is not transcriptionally but rather post-transcriptionally regulated. There is no knowledge yet, either on the regulation of *dim2* in *T. reesei* or on its action in the DNA methylation mechanism. In *N. crassa*, Dim5 acts as a histone H3 lysine methyltransferase (specifically, it trimethylates lysine 9) and was suggested to direct which cytosines will be methylated. The localization and action of Dim5 depend on the multiprotein complex DCDC (DIM-5/-7/-9, CUL4/DDB1 complex) [48]. Further, it was shown that the Heterochromatin protein 1 (HP1) recognizes the H3K9me3 marks and recruits Dim2 via direct protein–protein interaction [49]. To better understand how DNA methylation is regulated in *T. reesei* and which are the key players involved in the mechanism of DNA methylation, further in-depth studies are necessary. However, this study demonstrated, for the first time, the impact of the putative DMT Dim2 on cellulase expression.

The finding in this study that the induction of hypomethylation reduces the occurrence of the non-cellulase-producing phenotype enforces the assumption that DNA methylation plays a role in the mechanism behind the spontaneous loss of cellulase productivity and could be a starting point to increase strain stability. The examination of two different cellulase-productive strains, Iogen-M4 and Iogen-M10, revealed a strong difference in the reduction in the extent of degeneration as a response to hypomethylation. While hypomethylation reduced the degeneration of Iogen-M4 almost entirely, it had a weaker effect on the more productive strain Iogen-M10 (compare Figure 1). At first glance, one could argue that the differently pronounced effect of hypomethylation is correlated with the productivity of the strain and eventually also to a likely increase in secretory stress. However, whether and which connection of DNA methylation to productivity and related secretion stress exists is difficult to assess with the present results. For example, the DNA methylation pattern on sophorose does not follow this consideration. The global DNA methylation in QM6a is higher than in Rut-C30 and Iogen-M4 and equal to Iogen-M10, even though QM6a is, by far, the less productive strain. Also, Iogen-M10 has the highest DNA methylation under non-inducing conditions and even lower DNA methylation in the course of cellulase induction (sophorose). From the latter considerations, one could deduce that reduced DNA methylation is necessary for cellulase expression.

An alternative consideration for the observed differences in the effectiveness of hypomethylation for a reduction in (cel-) occurrence is an eventual dependence on the degree of DNA methylation. While the productive Iogen-M4 has a low level of DNA methylation, Iogen-M4 (cel-) has the same amount of methylated DNA as the productive Iogen-M10 under cellulase-inducing conditions (compare Figure 3). Given this, we would not correlate a certain level of methylation with a specific level of cellulase productivity, but we suggest a relation between the change in the methylation degree and the conditions favoring cellulase production (productive versus degenerated strains and inducing versus non-inducing carbon sources/compounds). Given that DNA methylation is a starting point in the degeneration phenomenon, it is likely that a strain with a lower initial DNA methylation level that is subjected to hypomethylation is less prone to degeneration. This could be the reason why hypomethylation works better in Iogen-M4, and the increased level of DNA methylation in Iogen-M10 could be the reason why hypomethylation has not such strong effect in reducing the rate of degeneration. Taken together, this reasoning suggests that a reduced DNA methylation level of a strain reduces its tendency to degenerate.

## 5. Conclusions

This study revealed a connection between the strain degeneration phenomenon in *T. reesei* and an increased level of global DNA methylation. An important finding was that artificial hypomethylation decreases the occurrence of the non-producing population to a strain-dependent extent. A second important finding was that inducing hypomethylation in a (cel-) strain recovered cellulase activity and partly reverted the degeneration phenomenon. However, the deletion of the putative DMT Dim2 did not release strains from degeneration in the long term. Therefore, for a comprehensive understanding of the connection of DNA methylation and strain degeneration, further studies are required. Exploring the molecular mechanisms causing DNA methylation might offer targets for preventing the loss of cellulase expression in *T. reesei* strains.

## Figures and Tables

**Table 1:** *T. reesei* strains used in the study.

Strain Name	Origin	Public Availability	Cellulase Expression Capacity	Protein Titer (g/g Biomass) ^1^	Sensitivity to Carbon Sources	Reference
QM6a	Wild-type isolate	Yes	Low	1	Sensitive to CCR ^3^	[32]
Rut-C30	3 rounds of RM ^2^ from QM6a	Yes	Moderate	12	Partial release of CCR due to *cre1* truncation	[31]
Iogen-M4	1 round of RM from Rut-C30	No	High	25	Strong release of CCR, full length *cre1* is missing	[7]
Iogen-M10	3 rounds of RM from Rut-C30	No	Very high	40	Strong release of CCR, full length *cre1* is missing	[8]

^1^ The protein titers were reported by Martzy et al., 2021 [8]. ^2^ RM, random mutagenesis. ^3^ CCR, carbon catabolite repression.

## Data Availability

The original contributions presented in the study are included in the article; further inquiries can be directed to the corresponding author.

## Supplementary Materials

The following supporting information can be downloaded at: https://www.mdpi.com/article/10.3390/microorganisms13030584/s1, Figure S1: Plasmid maps of pTSΔdim2_hph (left) and pTSΔrid1_hph (right) for the deletion of *dim2* and *rid1*. and Figure S2: Genotypic verification of the *dim2* and *rid1* deletion strains. After three rounds of homokaryon selection, the genotype of five putative Δ*dim2* and four putative Δ*rid1* strains was tested by PCR. Left: Two sets of primers were used to determine (i) the integration of the hygromycin marker in the correct locus of *dim2* or *rid1* and (ii) any presence of the wild-type (WT) band to check genetic homogeneity. Right: Image of the agarose gel electrophoresis of obtained PCR fragments. The 0.8% gels were run at 80 V for 45 min and the 1 kB DNA Ladder Plus GeneRuler (Thermo Fisher Scientific) was used as a marker (M). The orange and yellow circled lanes highlight the candidate strains that showed the correct genotype for the *dim2* or *rid1* deletion and were used for this study. Table S1: Oligonucleotides used in the study.
