# Supplementary material for: The Impact of DNA Methylation in Trichoderma reesei on Cellulase Production and Strain Degeneration
Source: Microorganisms. 2025 Mar 4;13(3):584. doi: 10.3390/microorganisms13030584 (PMC11946570; doi:10.3390/microorganisms13030584)
Supplement: Supplementary file 1 [file microorganisms-13-00584-s001.zip › microorganisms-3500693-supplementary.pdf]

Supplementary Material

Table S1. Oligonucleotides used in the study.

Name	Sequence (5′-3′)	Usage
TS99_rid1(NotI)	TAAGCGGCCGCGGAGAATTGCAGCCA CATCA	5′Flank rid1
TS101_rid1(BamHI)	TGTGGATCCCTAATGCCTCCCATTTGA	3′Flank rid1
TS100_rid (BamHI)	GTCGGATCCTTCTCGAGGTTGGGGAA GAA	5′Flank rid1
TSI02_rid1	CCTCCTGTGAGGGAAACCTG	3′Flank rid1
TS110(drid1)	CATTCTAGATGTCCTCTCGGATGATGA TCG	Deletion rid1 check WT band
TS111(drid1)	GACCCTGCAGGTCATGTAAGGTCAAC AACGGC	Deletion rid1 check WT band
TS112(drid1)	GACGACGGAGCGAAGAGAGT	Integration of hph marker in rid1 locus
TS113(drid1)	CCAACGTGGACAGCTGGATA	Integration of *hph* marker in *rid1* locus
RID1f2 (rutc30 7359)	GTTATGCATGATTCTTCACATCCAG	qPCR for *rid1*
RID1r2 (rutc30 7359)	GAGGTAGTGGGAACATCGATAAGAA	qPCR for *rid1*
TS54_NotI	TCTGCGGCCGCGTCGTCTTCATCACAA T	5′ Flank *dim2*
TS55	TCGCCAACCGAACTTCACCGAGA	5′ Flank *dim2*
TS56_BamHI	TCTGGATCCTGGGGTTCATATGCCTTG CT	3′ Flank *dim2*
TS57	CATGATAAAGCAGGGCCGAGTCA	3′ Flank *dim2*
TS18	CACATGCATTCAAAAGGGGTTCGGCC TCT	Deletion *dim2* check WT band
TS19	CACTCTAGAATGGATGCGTCATGGAG GACA	Deletion *dim2* check WT band
TS12	CCTAAGCAAAAGGCACAAGG	Integration of *hph* marker in *dim2* locus
Hph	GACCTGCCTGAAACCGAACTG	Integration of *hph* marker in *dim2* locus
DNMT1f	CTCTTCTGTGGAGGCGGTAACT	qPCR for *dim2*
DNMT1r	GTACGTGTGGATAGCCTTGCTG	qPCR for *dim2*

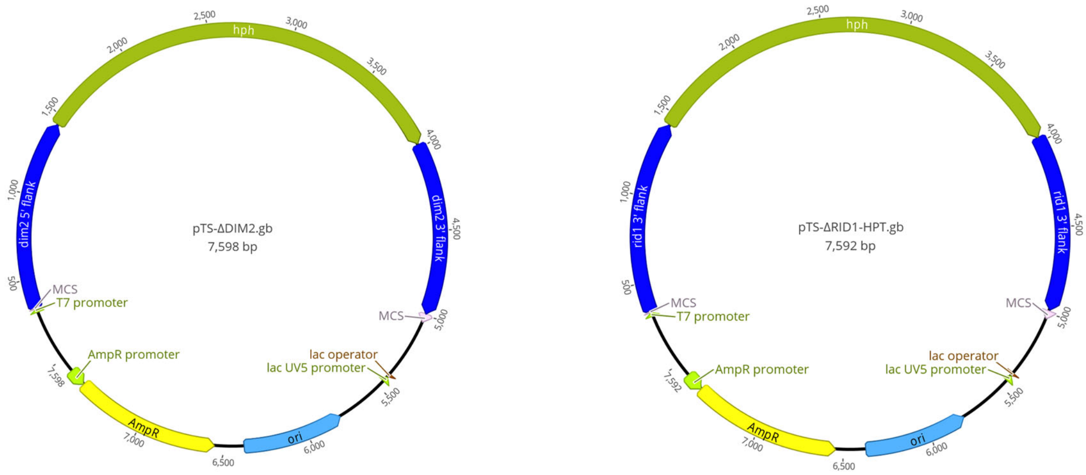

Figure S1. Plasmid maps of pTSΔdim2_hph (left) and pTSΔrid1_hph (right) for the deletion of *dim2* and *rid1*.

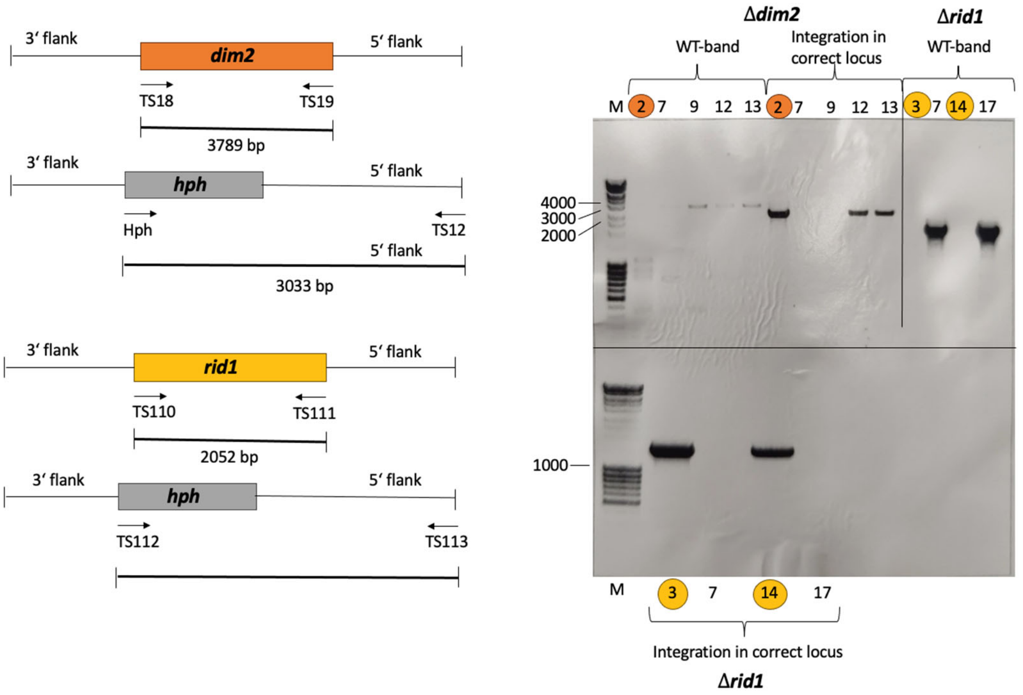

Figure S2. Genotypic verification of the *dim2* and *rid1* deletion strains. After three rounds of homokaryon selection, the genotype of five putative Δ*dim2* and four putative Δ*rid1* strains was tested by PCR. Left: Two sets of primers were used to determine i) the integration of the hygromycin marker in the correct locus of *dim2* or *rid1* and ii) any presence of the wild-type (WT) band to check genetic homogeneity. Right: Image of the agarose gel electrophoresis of obtained PCR fragments. The 0.8% gels were run at 80 V for 45 min and the 1 kB DNA Ladder Plus GeneRuler (Thermo Fisher Scientific) was used as a marker (M). The orange and yellow circled lanes highlight the candidate strains that showed the correct genotype for the *dim2* or *rid1* deletion and were used for this study.
